# Inferring neural circuit properties from optogenetic stimulation

**DOI:** 10.1371/journal.pone.0205386

**Published:** 2018-10-26

**Authors:** Michael Avery, Jonathan Nassi, John Reynolds

**Affiliations:** 1 Salk Institute for Biological Studies, La Jolla, CA, United States of America; 2 Inscopix, Palo Alto CA, United States of America; Georgia State University, UNITED STATES

## Abstract

Optogenetics has become an important tool for perturbing neural circuitry with unparalleled temporal precision and cell-type specificity. However, direct activation of a specific subpopulation of neurons can rapidly modulate the activity of other neurons within the network and may lead to unexpected and complex downstream effects. Here, we have developed a biologically-constrained computational model that exploits these non-intuitive network responses in order to gain insight into underlying properties of the network. We apply this model to data recorded during optogenetic stimulation in the primary visual cortex of the alert macaque. In these experiments, we found that optogenetic depolarization of excitatory neurons often suppressed neuronal responses, consistent with engagement of normalization circuitry. Our model suggests that the suppression seen in these responses may be mediated by slow excitatory and inhibitory conductance channels. Furthermore, the model predicted that the response of the network to optogenetic perturbation depends critically on the relationship between inherent temporal properties of the network and the temporal properties of the opsin. Consistent with model predictions, stimulation of the C1V1_TT_ opsin, an opsin with a fast time constant (tau = 45 ms), caused faster and stronger suppressive effects after laser offset, as compared to stimulation of the slower C1V1_T_ opsin (tau = 60ms). This work illustrates how the non-intuitive network responses that result from optogenetic stimulation can be exploited to gain insight regarding network properties that underlie fundamental neuronal computations, such as normalization. This novel hybrid opto-theoretical approach can thus enhance the power of optogenetics to dissect complex neural circuits.

## Introduction

Optogenetics has provided neuroscientists with the ability to perturb neural circuits with high temporal fidelity and cell-type specificity. This has led to substantial progress in understanding the neural circuit mechanisms underlying memory, perception, cognition and complex behavior [1–6]. Such experiments typically involve stimulating a subset of neurons and measuring behavioral changes and/or monitoring electrophysiological changes in a single local or downstream brain region. Although significant advances have been made using this approach, it is difficult to characterize the full set of feed-forward and feed-back effects that result from stimulating a subset of neurons in a particular area, which complicates the interpretation of the relationship between optogenetic activation and electrophysiological and behavioral changes.

**Fig 1:**
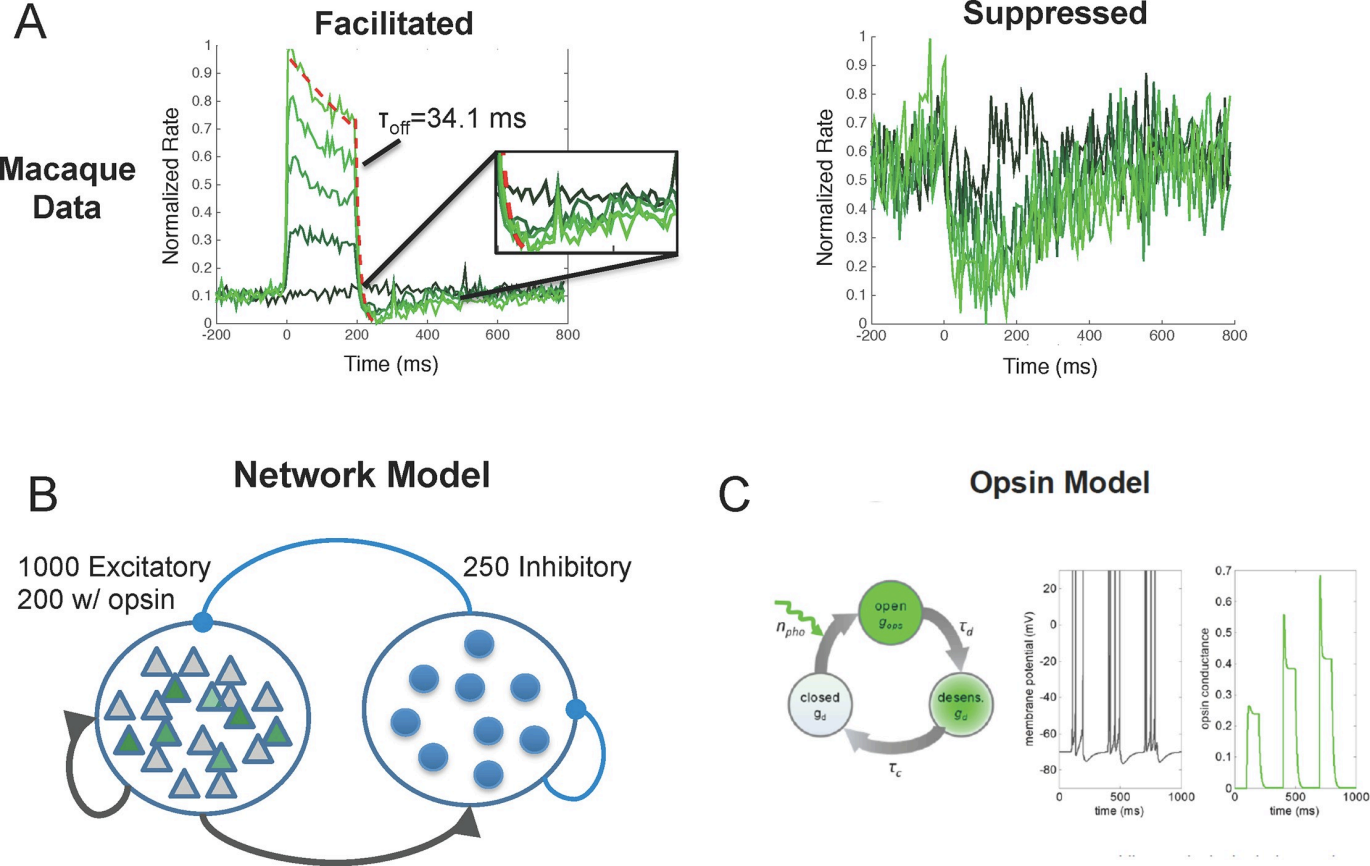
Recorded data from macaque V1 and model details. **A)** During laser stimulation, facilitated neurons had a fast onset response followed by a transient to sustained response profile similar to that seen following visual stimulus presentation. Immediately following laser offset, facilitated neurons in macaque V1 were quickly suppressed beyond their baseline firing rate for >100ms (left). The temporal response profile of suppressed neurons (right) reached peak suppression at ~100 ms after stimulation. After laser offset, the modeled and recorded neurons remained suppressed for ~200 ms and slowly returned to baseline. **B)** The modeled network is a recurrent neural network of 1000 excitatory and 250 inhibitory neurons and contains excitatory-excitatory, excitatory-inhibitory, inhibitory-excitatory, and inhibitory-inhibitory connections. Of the excitatory neurons, 200 contained the modeled opsin. **C)** The opsin conductance was modeled using a 3-state model, which includes an open, desensitized, and closed state. Increasing the number of photons (simulating different light levels) opens conductance channels (transient state) after which they become desensitized (sustained stated). When the light is turned off, conductance channels move to a closed state and the conductance decays to zero (left). A single simulated neuron and opsin conductance (right). As the opsin conductance increases, it drives the cell to fire at higher rates.

**Fig 2:**
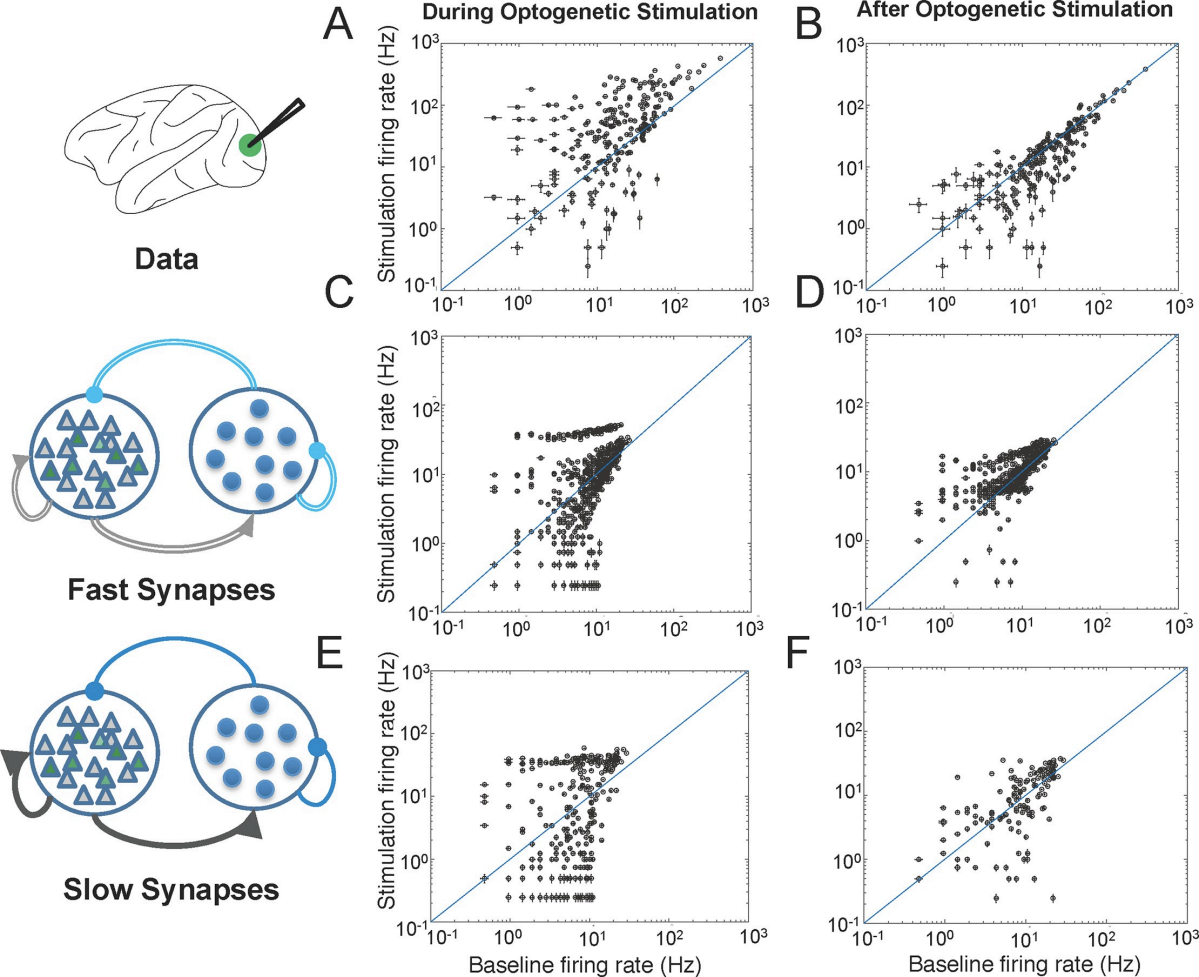
Modeling changes in mean responses. **A,B)** Mean responses from the data and simulation during optogenetic stimulation (Fig 2A; 0-200ms following laser onset) and immediately following stimulation (Fig 2B; 10–210 milliseconds following laser offset). (top) From the macaque data, a total of 249 units were recorded from two animals (142 from Monkey A, 107 from monkey M). Of these, 137 units were facilitated (55 single units, and 82 multi-units) and 23 units were suppressed (22 single units, 1 multi-unit) during optogenetic stimulation. Of these 160 units, 62 (42 single units, 20 multi-units) were also suppressed for the 10–210 milliseconds following laser offset (right). **B-E)** We developed two models, one that included fast excitatory and inhibitory synapses with τ_c_ = 5–10 ms, and one that included slow synapses, with τ_c_ = 100–200 ms. We tuned these models to capture the mixture of facilitative and suppressive effects seen during optogenetic stimulation as well as the suppressive effect across the population seen following optogenetic stimulation.

We have used optogenetics to investigate the normalization computation in macaque primary visual cortex (V1) [7]. We found that optogenetic depolarization of excitatory neurons can lead to a mixture of facilitative and suppressive effects in the network as has been shown previously [8], as well as a strong suppression of activity immediately following stimulation offset (Figs 1A, 2A and 2B). These suppressive effects were consistent with a model in which excitatory neuronal activation recruits a strong inhibitory response via indirect activation of inhibitory interneurons. These changes in neuronal responses were well captured by a normalization model in which increased excitatory drive is accompanied by divisive inhibition [9]. Optogenetic stimulation of cortical circuits can thus lead to complex changes in neuronal activity that are governed by feed-forward and feedback connections and local synaptic properties of the network.

To better understand this optogenetic-mediated suppression, we developed a spiking neural network that incorporates a biologically-realistic opsin conductance model. We applied this to better understand the normalization computation [10–13] and to gain insight into the effects that optogenetic stimulation has on cortical circuits. We found that our model’s response to optogenetic stimulation was strongly dependent upon synaptic strength and dynamics as well as properties of the opsin. Specifically, we found that slow synaptic dynamics were important for suppressive effects seen in the macaque and, therefore, could govern normalization computations seen in vivo. Our model was also able to correctly predict the suppression profile for a network expressing an opsin with faster kinetics. We thus conclude that optogenetic stimulation leads to a complex response pattern that is determined by both the temporal properties of the opsin and intrinsic properties of the circuit and that understanding this relationship can enhance our ability to use optogenetics to gain insight into the underlying circuit and circuit-level computations.

## Results

### Modeling the mean responses of optogenetic stimulation in macaque V1

Optogenetic depolarization of excitatory neurons in macaque V1 leads to modulation of baseline firing rates amongst a mix of directly and indirectly activated neurons in the local network. Interestingly, we observed facilitation and suppression of neuronal responses during stimulation, followed by a period of suppression after laser offset (Figs 1A, 2A and 2B), despite the fact that we were only stimulating excitatory neurons. We hypothesized that this suppression seen during laser stimulation was mediated by strong feedback inhibition in the network and that the after-suppression is mediated by the dynamics of receptors in the synapse.

To determine how feedback inhibition and synaptic dynamics influence the mean responses during and following optogenetic stimulation, we developed neural network models that incorporated opsin into excitatory neurons, included strong feedback inhibition, and had either fast or slow synaptic dynamics (Fig 1B and 1C, Methods). Each network was tuned to match the facilitative and suppressive mean response effects seen in vivo during laser stimulation (Fig 2A) and immediately following stimulation (Fig 2B). We classified model neurons as facilitated or suppressed using the same metrics as we used for the neuronal data set.

A network with strong feedback inhibition mediated by strong exc->inh and inh->exc connection weights was sufficient to produce a mixture of facilitative and suppressive responses at the population level regardless of synaptic dynamics during simulated laser stimulation (Fig 2C and 2E). However, as discussed below, the proportion of the population exhibiting facilitation during stimulation vs post-stimulation suppression did depend on the dynamics at the synapse.

In the network with fast synaptic dynamics (τ_c_ = 5-10ms) we found that 593 of the 1250 units (47%) were significantly facilitated (55% facilitated in vivo) and 303 of the units (24%) were significantly suppressed (10% suppressed in vivo) during optogenetic stimulation. Of these 896 facilitated and suppressed units, 171 units (19%) were suppressed (39% suppressed in vivo) for the 10–210 milliseconds following laser offset (Fig 2F).

A network with slow synaptic dynamics (τ_c_ = 100–200 ms), however, was better able to account for the suppressive effect observed immediately following laser stimulation (Fig 2D and 2F). Of the 1250 neurons in the network that incorporated slow synapses, 420 of the units (34%) were significantly facilitated (55% facilitated in vivo) and 250 of the units (25%) were suppressed (10% suppressed in vivo) during optogenetic stimulation. Of these 670 facilitated and suppressed units, 350 units (52%) were also significantly suppressed (39% suppressed in vivo) for the 10–210 milliseconds following laser offset (Fig 2F). We believe that the number of suppressed units in the model was greater than what was found in the macaque due to the difficulty in identifying neurons with a high baseline firing rate that show suppression due to the laser.

The network with slow synapses, therefore, more accurately accounted for the after suppression following laser offset whereas the network with fast synapses more accurately accounted for the number of facilitated neurons seen during stimulation. The differences that were seen in the mean responses between networks with fast and slow synapses led us to more closely investigate how synaptic dynamics influence the full temporal response profiles of neurons to optogenetic stimulation as discussed in the following section.

### Modeling the temporal dynamics of optogenetic stimulation in macaque V1

**Fig 3:**
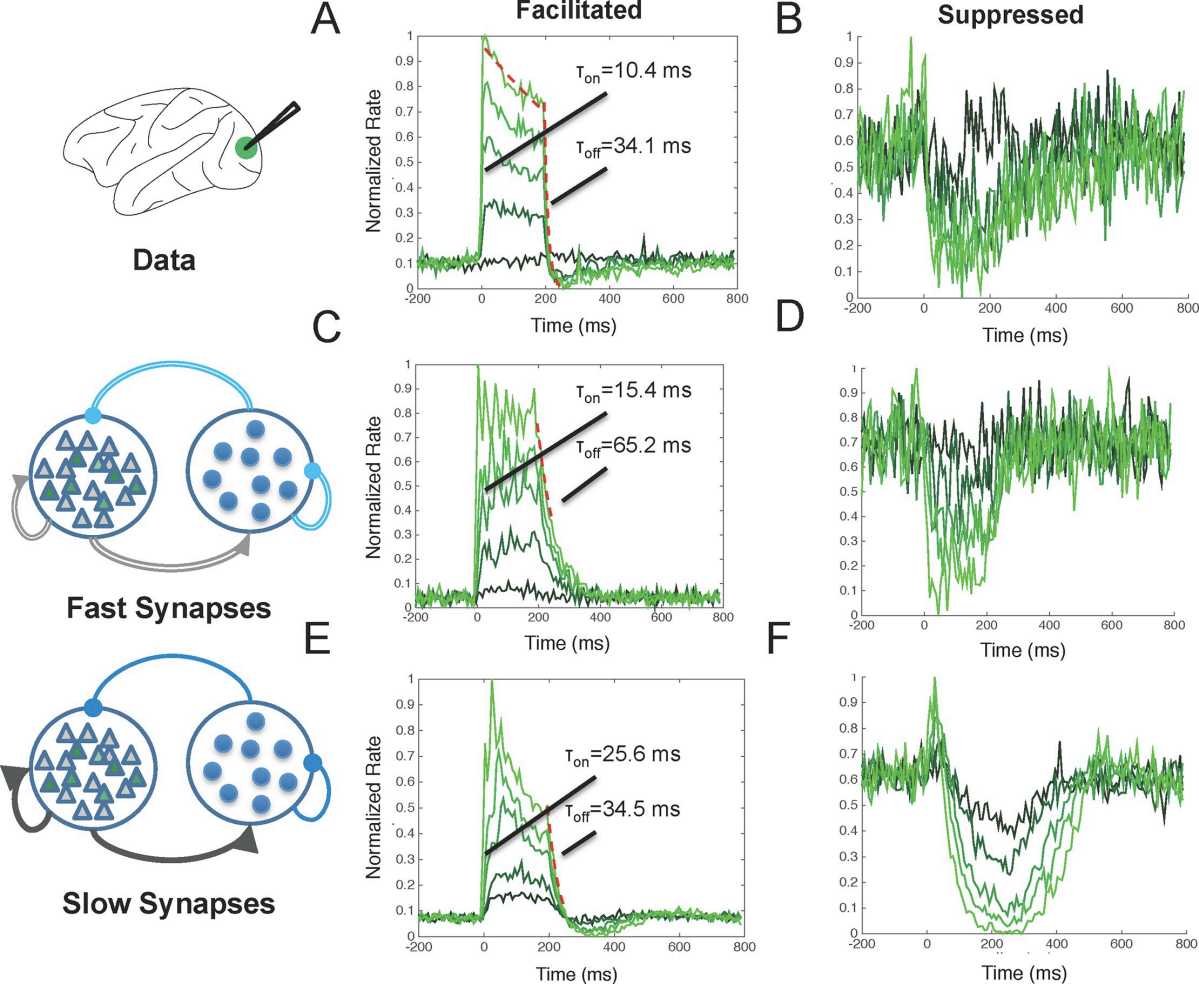
Model comparison with the full temporal response profile seen in the data. **A,B)** Temporal response profile during optogenetic stimulation for facilitated (Fig 3A) and suppressed (Fig 3B) neurons in macaque V1. **B-E)** The full response profile of the models developed in Fig 2A, including a model with fast synapses (Fig 3B and 3C) and a model with slow synapses (Fig 3D and 3E).

In order to gain insight into how synaptic dynamics influence the full temporal response profile of the network to optogenetic stimulation, we compared the full temporal response profile of facilitated and suppressed neurons recorded in macaque V1 with our simulated data. During laser stimulation, facilitated neurons in macaque V1 had a fast onset response (mean = 10.4 ms) followed by a transition from this transient onset to a sustained response profile, similar to that seen following visual stimulus presentation with high contrast stimuli (Fig 3A).

We saw this same response profile in facilitated neurons in the simulated data (Fig 3C and 3E) including a quick response onset (mean = 15.4 ms for fast synapses and mean = 25.4 ms for slow synapses) and a transient to sustained response profile. This result was found despite the simplicity of the model and the fact that we did not tune the model to explicitly match the onset time distribution. Interestingly, the response onset latency of the data was a closer match with the onset latency of the model that incorporated fast synaptic dynamics (τ_c_ = 5–10 ms) when compared with the model that incorporated slow synaptic dynamics. The transient-to-sustained ratio of the data, however, matched closer with the transient-to-sustained ratio of the model that incorporated slow synaptic dynamics. The mean transient to sustained ratio for the network with fast synapses was approximately 1.0, indicating that there was no transient to sustained response. The transient to sustained ratio for the network with slow synapses, however, was 1.6. Together, these results suggest that slow synaptic feedback mediates the shape of the transient to sustained response profile and fast, feedforward inhibition mediates the onset response latency.

Immediately following laser offset, facilitated neurons in macaque V1 were quickly suppressed (median τ_network_ = 34.1 ms) beyond their baseline firing rate for >100ms (Fig 3A). We quantified the magnitude of this suppression as the normalized distance between the mean baseline rate and post stimulation rate. Facilitated neurons in the model with fast synapses showed a slower return to baseline (median τ_network_ = 65.2 ms) and no suppression following laser offset (Fig 3C). Facilitated neurons in the model with slow synapses, however, showed fast suppression (median τ_network_ = 34.5 ms) following laser offset (Fig 3E) and strong suppression with a mean normalized magnitude of suppression equal to 0.06.

The temporal response profile of suppressed neurons seen in the macaque during laser stimulation (Fig 3B) was also found in the simulated data (Fig 3D and 3F). For neurons showing suppression during stimulation, both the model and data had a smoother response profile, lacking the transient peak seen in facilitated neurons. After laser offset, recorded neurons remained suppressed and then slowly returned to baseline after 200–300 ms (Fig 3B). We see in comparing Fig 3D and 3F that the slow synaptic time constants were critical for replicating the slow return to baseline seen in the data at laser offset in suppressed neurons. To quantify this, we compared the mean normalized magnitude of suppression in the two networks from 100–200 ms following laser offset and found the network with fast synapses to have significantly weaker suppression than the network with slow synapses (mean suppression (fast) = 0.07, mean suppression (slow) = 0.1; p < 0.05). We thus conclude that the fast and slow networks differentially responded to laser offset and that the network with slow synaptic dynamics was able to better capture the laser offset response of both facilitated and suppressed neurons when comparing with data from the macaque.

### Temporal dynamics of neuronal responses to laser offset depend on properties of the network

**Fig 4:**
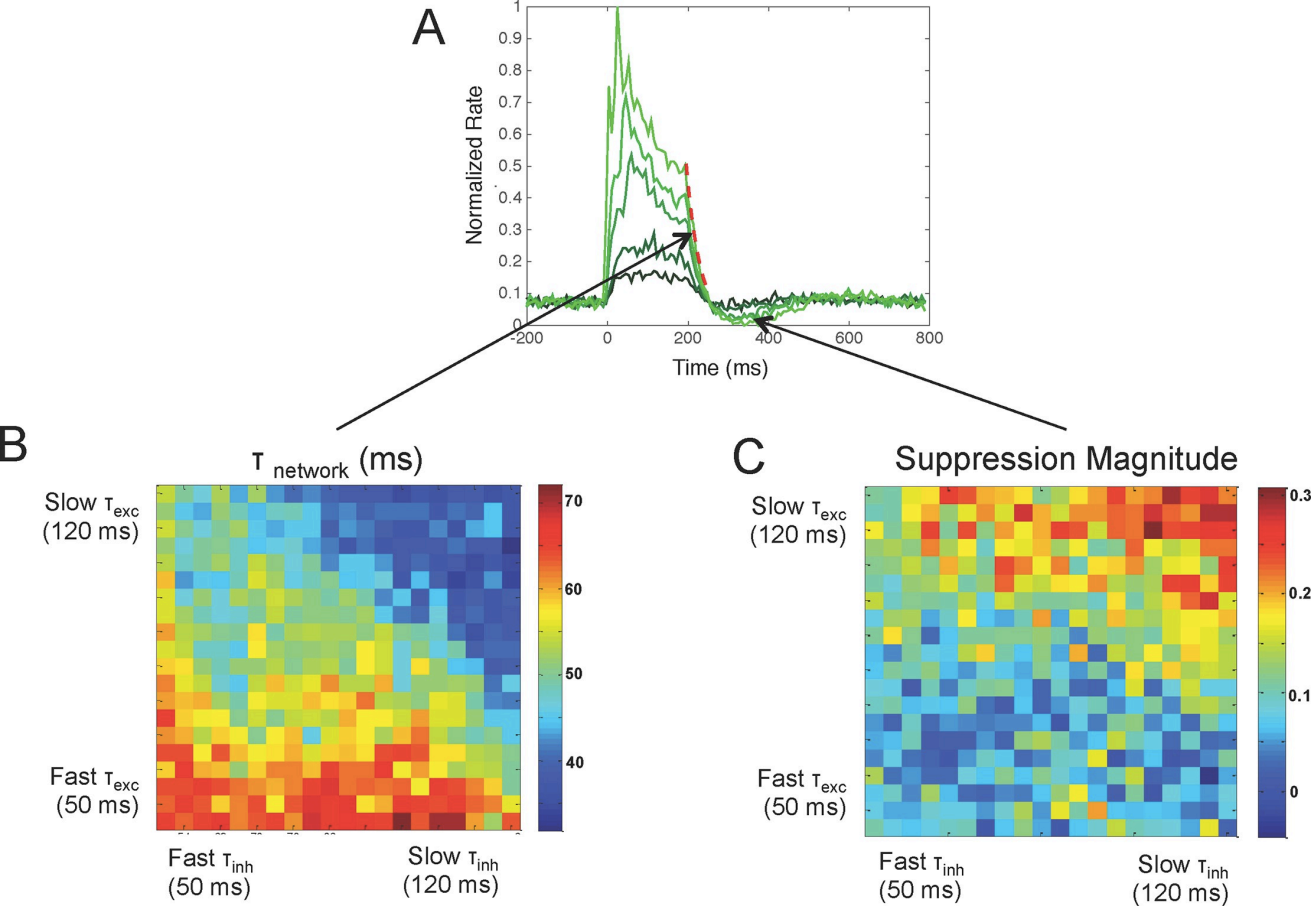
After-suppression depends on local network properties. A) Shown is an example response profile of facilitated neurons to optogenetic stimulation. We modeled the decay to baseline with an exponential function and computed the absolute magnitude of suppression relative to baseline and used these values for Fig 4B and 4C. B) We found that the decay to baseline was faster when excitatory and inhibitory time constants increased (upper right) and slower when excitatory and inhibitory time constants decreased (lower left). C) The magnitude of suppression was also greater for larger excitatory and inhibitory time constants (upper right) compared to small excitatory and inhibitory time constants (lower left).

The temporal profile of suppression following laser offset appears to be influenced by synaptic dynamics as discussed above. In order to more systematically investigate this, we simultaneously varied the t_exc_ and t_inh_ parameters in the model and measured their effects on the opsin-expressing neurons in the network (Fig 4). Specifically, we measured how fast opsin-expressing model neurons decayed to baseline firing rates when the laser was extinguished and how strongly they were suppressed below the baseline response. The decay to baseline time constant (τ_network_) was modeled using an exponential function and the magnitude of suppression was taken as the distance between the baseline and suppressed response in normalized firing rates.

We found that τ_network_ is inversely correlated with t_exc_ and t_inh_ in the model. As the excitatory and inhibitory time constants increase, the opsin-containing neurons decayed to baseline at a faster rate. In particular, as t_exc_ and t_inh_ increase (> 100 ms), τ_network_ decreases to 30 ms (Fig 4, left). As t_exc_ and t_inh_ decrease (< 20 ms), however, τ_network_ increases to 65 ms, which is close to the time constant of the opsin (Fig 4). This suggests that τ_network_ is determined by the opsin only if the excitatory and inhibitory time constants are less than the opsin off time constant. Therefore, the τ_network_ response of the opsin containing neurons is not determined by the opsin, excitatory, or inhibitory time constants alone, but, rather, a relation between all three of these time constants.

The magnitude of suppression was also dependent upon the excitatory and inhibitory synaptic time constants of the network. We found that as excitatory and inhibitory time constants increase, the magnitude of suppression also increases (Fig 4). In particular, as t_exc_ and t_inh_ increase (> 100 ms), the normalized magnitude of suppression increases to 0.3 (Fig 4, right). This is due to the strong inhibitory conductances that have built up on excitatory neurons combined with their slow decay to baseline relative to the opsin and excitatory conductances. As t_exc_ and t_inh_ decrease (< 20 ms), however, the magnitude of suppression approaches zero as there is no longer an after-suppressive effect. The trend shown in Fig 4 suggests that slow synaptic dynamics are critical for after-suppression, emphasizing the fact that slow receptors likely played a significant role in regulating the suppressive effects that were seen in macaque V1.

### Temporal dynamics of neuronal responses to laser onset and sustained laser stimulation depend on properties of the network

**Fig 5:**
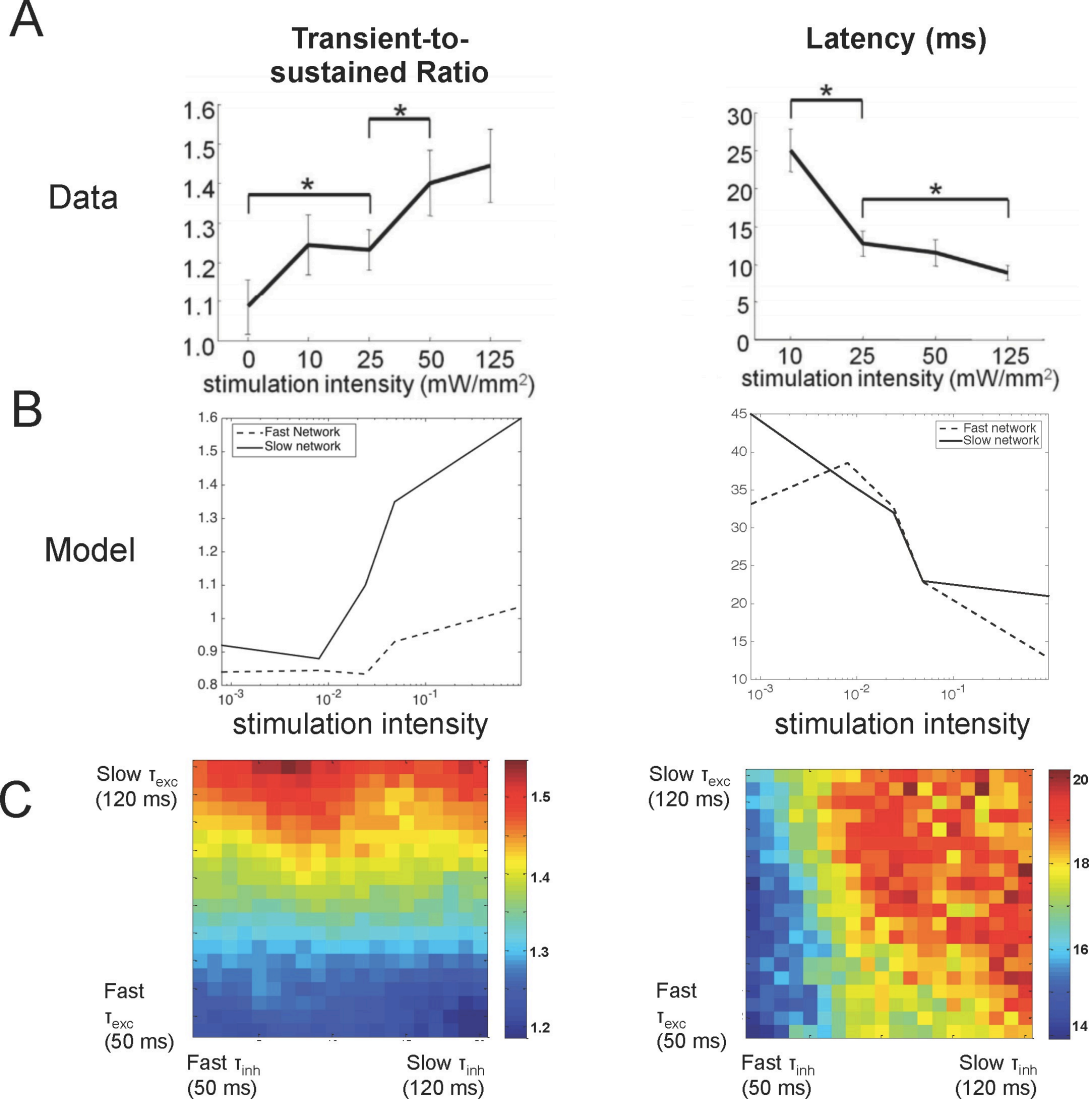
Intensity and synaptic-dependent changes in latencies and transient-to-sustained ratios. A) In the macaque, the ratio of the transient peak to sustained response increased as a function of laser intensity (left) and latency of neuronal responses decreased (right). B) The model that incorporated slow synapses also showed an increase in the transient to sustained ratio as a function of stimulation intensity (left), consistent with the results in the macaque. The model with fast synapses, however, could not faithfully reproduce the transient to sustained ratio found in the macaque, but more accurately accounted for the decrease in latency seen with laser intensity (right). C) The model suggests that the transient-to-sustained ratio and latency are dependent upon synaptic time constants. In particular, the transient-to-sustained ratio increases as a function of the excitatory time constant (left) and the latency increases as a function of the inhibitory time constant (right). These simulations were performed at the highest simulated intensity level.

Optogenetic stimulation of macaque V1 produced intensity-dependent responses that mimic changes seen during visual stimulation. In particular, with increased laser intensity, as with visual contrast, V1 responses exhibited a decrease in response onset latency and increase in the ratio of the transient onset response to the sustained response (transient-to-sustained ratio Fig 5A). The kinetics of the opsin, however, also show intensity dependent changes in the transient and sustained opsin currents, making it difficult to know whether these effects resulted from a property of the opsin or a property of the network. Given the established relationship between synaptic time constants and the magnitude and length of post-stimulation suppression shown in the previous sections, we hypothesized that intensity-dependent response changes seen with optogenetic stimulation are also dependent upon network properties and not simply properties of the opsin alone.

To determine whether or not the fast and slow networks can match changes in responses we saw with varying laser intensity in vivo, we varied the *n* parameter in Eq 6, which controls the number of photons that activated the simulated opsin. We, indeed, show that by varying this parameter, the simulated network that incorporated slow synaptic dynamics qualitatively matched the intensity-dependent responses seen in the macaque. Without any additional tuning, Fig 5B shows that with increasing intensity, there is a decrease in response latency and an increase in the transient-to-sustained ratio in the network with slow synapses. This is consistent with results we saw in the macaque as well as results found in a recent paper that measured the change in peak and steady-state current into a cell as a function of stimulation intensity [14]. The network with fast synapses, however, did not show a transient-to-sustained ratio >1 as was seen in vivo, but demonstrated a fast onset latency (12.4 ms) with the highest intensity, which better matched the 10.4 ms latency seen in the macaque at highest laser intensity.

We varied network parameters governing synaptic dynamics to test whether or not the transient to sustained ratio and latency vary systematically with synaptic parameters as we found earlier with the after-suppressive effect following laser offset. We found that the response latency and transient-to-sustained ratio do, in fact, depend on the excitatory and inhibitory synaptic time constants of the network. Response latency is directly dependent upon the time constant of inhibition, so that a faster time constant leads to shorter response latencies (~14 ms) and a slower time constant of inhibition leads to longer latencies (~20 ms), when stimulating at the highest simulated laser intensity (Fig 5C, right). The transient-to-sustained ratio, on the other hand, directly depends on the time constant of excitation (Fig 5C, left. The transient to sustained ratio becomes larger and more apparent as the time constant of excitation increases. The model therefore suggests that the observed response latencies and transient-to-sustained ratios, which are built-in properties of the opsin itself, may, in fact, also depend on local network properties. Moreover, it suggests that the fast onset response seen in the macaque is dependent upon fast inhibition, but slow excitation, which leads to slow feedback inhibition, is important for driving responses during the sustained period of laser stimulation.

### Properties of opsin provide insight into network properties

Our simulations suggest that the temporal response of a network of neurons to optogenetic stimulation depends on local network properties. These simulations assumed the kinetics of the simulated opsin matched the C1V1 _T_ opsin used in the experimental recordings in the macaque. We hypothesized that the time constant of the opsin relative to the network was critical in determining the temporal response profile. To test this, we changed the time constant of the opsin in the model to be larger (τ_c_ = 80 ms) or smaller (τ_c_ = 40 ms) than the C1V1_T_ opsin and measured τ_network_ and post-stimulation suppression magnitude as a function of t_exc_ and t_inh_. We were interested to know whether or not changes in the opsin time constant would produce different suppression profiles.

**Fig 6:**
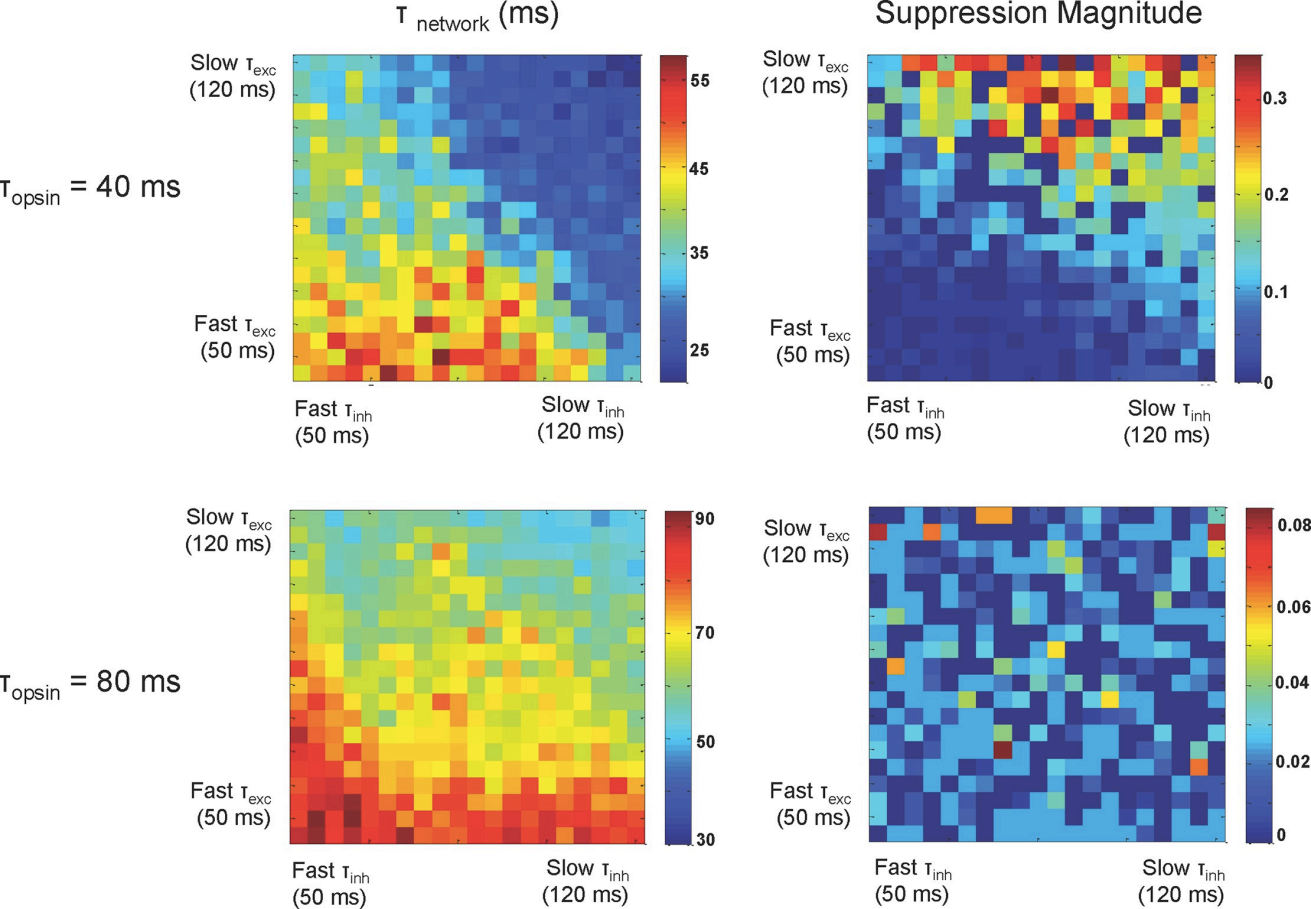
After-suppressive effects depend on the time constant of the opsin. These plots from network simulations depict how fast the network falls to baseline following laser stimulation (left) and the magnitude of suppression (right) as a function of the excitatory and inhibitory time constants. As in Fig 5, the model demonstrates a faster decay to baseline and stronger magnitude of suppression in networks that have larger excitatory and inhibitory time constants. Here, we show that the model also predicts that there should be stronger and faster suppression in the network with a faster opsin time constant (τ_c_ = 40 ms, top) compared to the slower opsin time constant (τ_c_ = 80 ms, bottom). In fact, when the time constant of the opsin is increased to 80 ms, the after-suppressive effect seen following laser stimulation is completely eliminated (bottom-right) no matter what the excitatory and inhibitory time constants of the network are.

We found that the time constant of the simulated opsin influences the strength and speed of the suppression following laser offset in our modeled network (Fig 6). A simulated opsin time constant of 40 ms and slow synaptic time constants resulted in strong after-suppression (Fig 6, top right) and fast off kinetics (Fig 6, top left). A simulated opsin time constant of 80 ms abolished after-suppression, regardless of synaptic time constants (Fig 6, bottom right), but still showed faster off-kinetics with slower synaptic time constants (Fig 6, bottom left). The model thus predicts that the use of an opsin with faster off kinetics should result in a faster and stronger suppression and an opsin with slower kinetics would result in little to no after-suppression.

**Fig 7:**
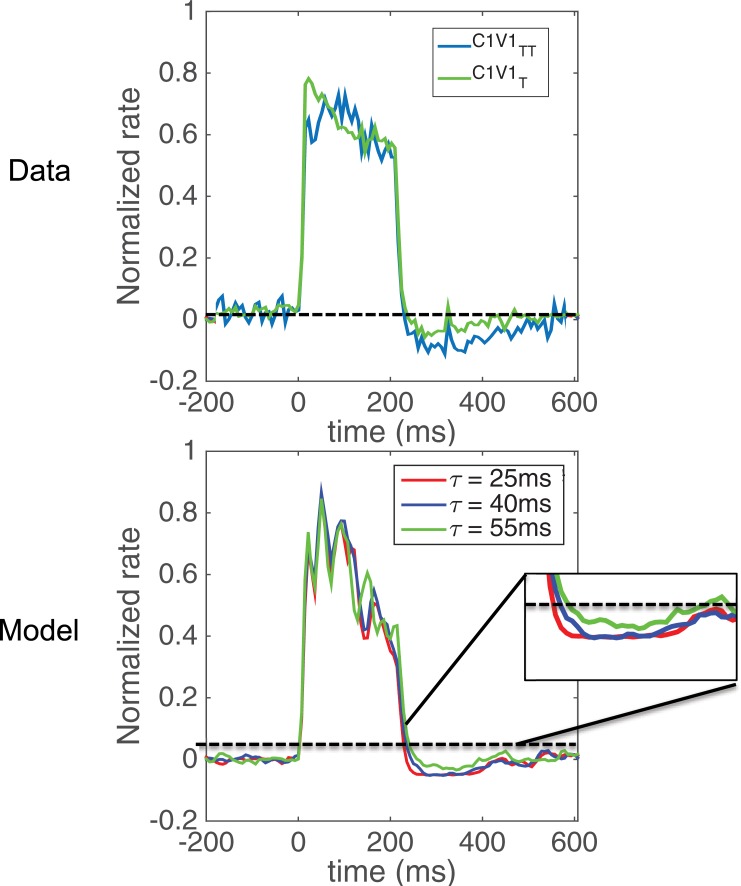
Experimental data from macaque primary visual cortex matches model prediction. By analyzing the C1V1 _TT_ (t _off_ = 45 ms) and C1V1 _T_ (t _off_ = 60 ms) temporal response profiles obtained from macaque V1, we see that the C1V1 _TT_ opsin shows higher magnitude (top) of suppression following laser offset. This is consistent with the model prediction (bottom).

We were able to test the first prediction by analyzing neuronal responses obtained from macaque V1 involving optogenetic stimulation of C1V1_TT_ opsin (t_off_ = 45ms, t_des_ = 75ms) and comparing it to stimulation of C1V1_T_ opsin (t_off_ = 60ms, t_des_ = 55 ms). The C1V1_TT_ data set included 16 facilitated neurons compared to the 249 neurons in the C1V1_T_ data set. As predicted, the C1V1_TT_ opsin shows a stronger after-suppressive effect than the C1V1_T_ data as well as a faster offset of response as we quantify below. This data obtained from the macaque (Fig 7, top) matches well with model predictions (Fig 7, bottom).

**Fig 8:**
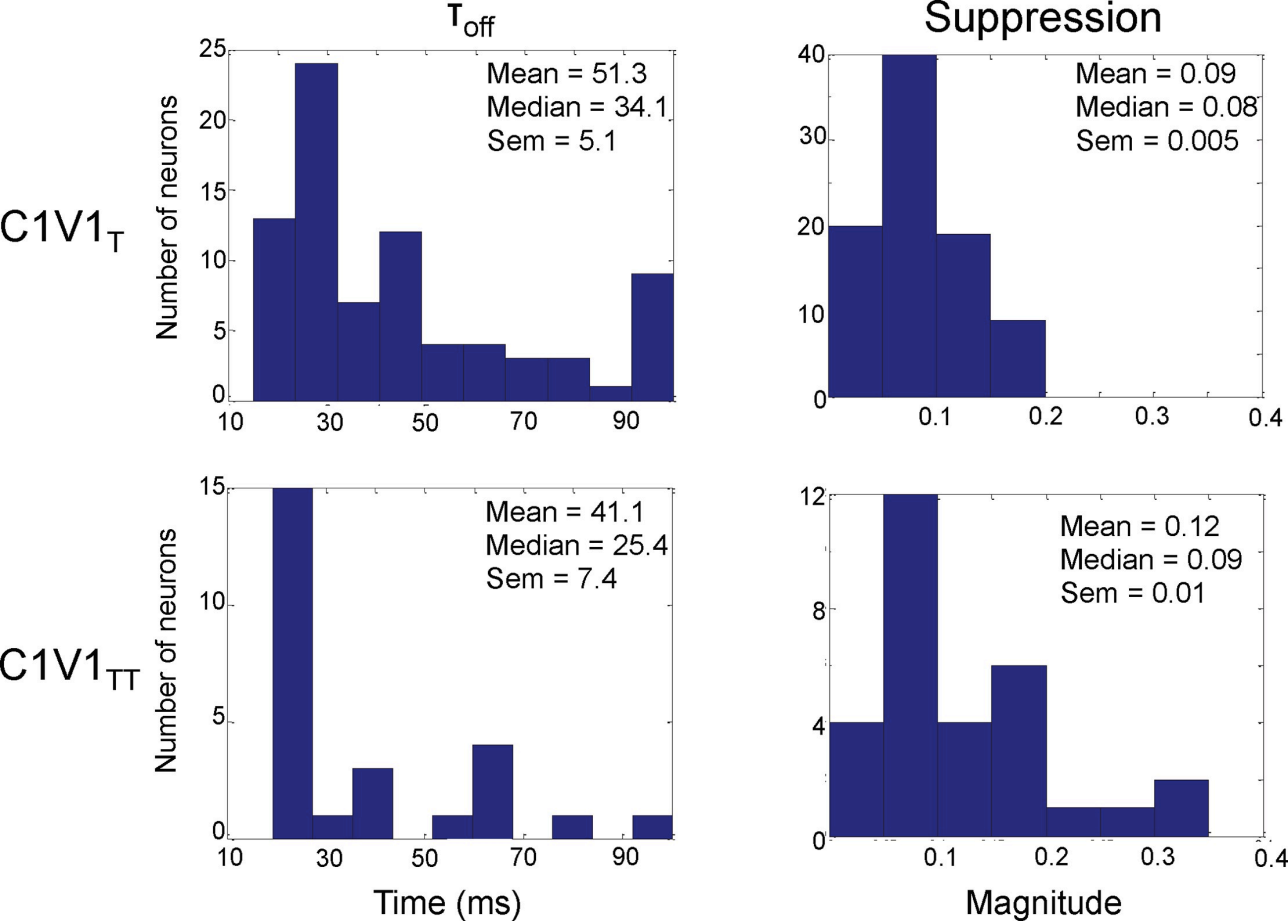
Magnitude and speed of suppression of neuronal responses to stimulation of C1V1 _TT_ and C1V1 _T_ opsins in macaque V1. Comparing the histograms of the off response and magnitude of suppression of the C1V1 _TT_ and C1V1 _T_ on a cell-by-cell basis, we see that the faster opsin (C1V1 _TT_) has a significantly faster off response (left) and a stronger magnitude of suppression (right) as predicted by the model.

As shown in Fig 8, the off responses of the C1V1 _TT_ data from macaque V1 (median = 25.4, standard error = 7.4) were smaller than the off responses of the C1V1 _T_ data (median = 34.1, standard error = 5.1) and the distribution of these two data sets was significantly different (Wilcoxon rank-sum test, p<0.05). As also predicted by the model (Fig 6), the magnitude of suppression was significantly larger (Wilcoxon rank-sum test, p<0.05) in the C1V1_TT_ data set (median = 0.09, standard error = 0.01) than the C1V1_T_ data set (median = 0.08, standard error = 0.005). For these analyses, we compared the full C1V1_TT_ data set with a subset of the C1V1_T_ data set that matched the baseline and stimulation rates of the C1V1_TT_ data set. This was done so that a fair comparison could be made between the C1V1 _TT_ data, which had only 16 neurons and high baseline firing rates, and the C1V1 _T_ data, which had 249 neurons and a much wider variety of baseline and stimulation rates. Given these results, we conclude that decreasing the time constant of the opsin relative to the network time constants causes a stronger and faster after-suppression following laser offset as predicted by our model.

## Discussion

We developed a neural network model that incorporates a simulated opsin conductance in order to better understand the suppressive effects observed experimentally when excitatory neurons in macaque V1 were optogenetically depolarized. We found that the synaptic dynamics of the network can strongly influence laser response-onset profiles, including latency and transient-to-sustained ratio, and laser-offset profiles, including off kinetics of the network and post-stimulation suppressive effects. In particular, strong and slow recurrent inhibition seems to be critical for the post-stimulation suppressive effects observed in macaque V1 as well as the transient-to-sustained ratio. Moreover, response latency and the transient-to-sustained ratio are positively correlated with inhibitory and excitatory synaptic time constants, respectively. Finally, the model predicted that decreasing opsin off kinetics would lead to faster and stronger suppression and this was verified by comparing data from the C1V1_T_ opsin and C1V1_T T_ opsin.

Taken together, these results suggest that the fast off response and strong suppression observed in the macaque are mediated by slow excitatory and inhibitory feedback signals such as NMDA receptors on GABAergic neurons and GABA-B receptors expressed on glutamatergic neurons. This would lead to a slow, sustained inhibition following laser offset. This would be consistent with non-fast spiking neurons, such as somatostatin neurons, being activated by stimulated excitatory neurons, which has been shown experimentally [15]. Somatostatin neurons have been shown to act on GABA-B receptors and suppress dendritic activity [16–18] and may play a role in normalization computations [19]. NMDA receptors, on the other hand, are thought to be involved in the initiation of the up state [20], feedback connections [21], attention [22], and oscillations [23]. NMDA receptors have also been shown to be localized presynaptically on excitatory neurons synapsing on somatostatin neurons [24], lending further support to the idea that NMDA and GABA-B receptors are mediating this feedback suppressive effect we and others have observed in vivo. It is important to note that, given the nature of the slow inhibitory feedback mediating the suppressive effects in our model, we would expect less pronounced suppression for shorter duration pulses and more pronounced suppression for longer duration pulses.

Our model, on the other hand, also demonstrates that short response latencies are realized through fast excitatory and inhibitory receptors in cortical circuits. This suggests that the initial response onset may be mediated by fast synaptic dynamics including AMPA and GABA-A receptors in vivo. The idea that initial responses are mediated by fast excitatory receptors whereas feedback is mediated by slow excitatory receptors has been shown recently [21]. Moreover, the model suggests a critical role for fast synapses in regulating response latency, suggesting these synapses play a role in response reliability as has also been recently found experimentally [25]. Cognitive processes such as attention, which influence response reliability and latencies [26–28], may thus act on fast excitatory and inhibitory conductances.

In the macaque, we found that optogenetic stimulation could suppress visually evoked responses as well. This suppression was well characterized by the normalization model in which excitatory responses are divisively suppressed by pooled neuronal activity in the network. Several theories exist suggesting that normalization may be achieved through (1) synaptic depression [29], (2) increases in excitatory and inhibitory conductances [30], or (3) inhibition [31]. Normalization-like responses have been shown experimentally to be mediated by somatostatin neurons [32] as well as cortical feedback [33]. Our model suggests that slow NMDA and GABA-B conductances are important for optogenetic-induced suppression and therefore may play a role in the normalization computation.

Given the conclusions that follow from these findings, it will be interesting to more deeply investigate the role that both fast and slow synaptic receptors play in modulating the dynamics of the network in spontaneous and stimulus evoked states, what role different neuronal types may play in this process, and how these receptors and neurons ultimately play a role in cognitive processes such as attention and perception. In the future, we believe that using models to more deeply understand the influence that optogenetic stimulation has on the brain will be informative given the non-local changes that are induced in the network.

## Methods

### Experimental procedures and data

All data used in this study and all experiments were previously published in Nassi et. al. 2015. Briefly, two macaque monkeys were implanted with a head post and chamber for awake, behaving primate experiments. A craniotomy and durotomy were made within the chamber and an artificial dura was implanted to provide visibility for electrode penetrations and allow for optical stimulation with light. We injected two animals with a lenti-CAMKII-C1V1_T_ virus at a single location for electrophysiological recordings and in a separate animal assessed the virus’ specificity for excitatory neurons using immunohistochemistry procedures. In one of the animals, we made a second injection of AAV5-CAMKII-C1V1_TT_ far from the original lentivirus injection, but still within V1. After allowing six weeks for opsin expression, electrophysiology and simultaneous optogenetic stimulation experiments commenced. Optogenetic stimulation was randomly interleaved at various light intensities and the effects of stimulation were compared with baseline firing rates. Optogenetic stimulation was delivered continuously for a duration of 200 ms every trial. Continuous depolarization for this time period was chosen to match visual stimulation, which was also 200 ms on trials that incorporated a visual stimulus.

Experimental and surgical procedures were approved by the Salk Institute Institutional Animal Care and Use Committee and conformed to NIH guidelines for the care and use of laboratory animals.

### Network model

The network model (Fig 1B) is comprised of a population of 1000 excitatory (E) neurons and 250 inhibitory (I) neurons, all recurrently connected. Connection strengths between E-E, I-E, E-I, and I-I neurons were set equal to 2.5x10^-1^ nA, 4.0x10^-1^ nA, 4.0x10^-1^ nA, and 1.0x10^-0^ nA, respectively. The probability of connectivity between all neuron groups was set to 10%. As will be explained below, these parameters were chosen so that the model would match the facilitative and suppressive effects that are observed in vivo with optogenetic stimulation. Both excitatory and inhibitory neurons were modeled using a leaky integrate and fire model:

$$C_{m}\dot{v}(t)=-g_{leak}(v(t)-V_{leak})-{I(t)}_{syn}—{I(t)}_{ops}-{I(t)}_{noise}$$ (1)

$${I(t)}_{syn}=g_{exc}(v(t)-V_{exc})+g_{inh}(v(t)-V_{inh})$$ (2)

$${I(t)}_{ops}=g_{ops}\left(v(t)-V_{ops}\right)$$ (3)

Where g_leak_ = 0.05 nA, V_leak_ = 0 mV, V_exc_ = 0 mV, V_inh_ = -70mV, and V_ops_ = 0 mV. g_ops_ was governed by the opsin Eqs (6–8). I_noise_ is the time-varying background noise governed by the following equation:

$$\tau_{n}\dot{I}(t)=-(I(t)-I_{0})+\sqrt{\tau_{n}}\sigma_{n}\eta (t)$$ (4)

Where τ_n_ = 2 ms, I_0_ = 0.1 nA, σ_n_ = 0.003 nA, and η(t) is Gaussian white noise. Excitatory and inhibitory synapses were conductance based and governed by the following equations:

$${\dot{g}}_{x}=-\frac{g_{x}}{\tau_{x}}+\sum_{j}\delta (t-t_{j})$$ (5)

where *x* = excitatory (exc) or inhibitory (inh) and τ_exc_ and τ_inh_ were picked from a uniform distribution from 150–160 ms unless otherwise noted.

### Opsin Model

Optogenetics involves stimulating an opsin protein that is embedded within a cell membrane using light of a particular wavelength. We adopted a model (Fig 1C) for channelrhodopsin developed by [34], which is able to account for the dynamics of membrane depolarization with light onset. Within the model, stimulation opens the channelrhodopsin protein, causing a sharp transient depolarization of the cell followed by a less depolarized sustained phase. The opsin conductance remains in the desensitized state until the light is turned off at which time it moves to the closed state. This model is governed by the following system of equations:

$${\dot{g}}_{ops}=n\beta_{c}-\frac{g_{ops}}{\tau_{d}}$$ (6)

$${\dot{\alpha }}_{d}=\frac{g_{ops}}{\tau_{d}}-\frac{\alpha_{d}}{\tau_{c}}$$ (7)

$${\dot{\beta }}_{c}=\frac{\alpha_{d}}{\tau_{c}}-n\beta_{c}$$ (8)

where g_ops_ is the total opsin conductance, which is governed by the desensitization rate α_d_ and close rate β_c_ of the channels. The time constants governing the desensitization and closing of the opsin channel are given by τ_d_ and τ_c_, respectively. The *n* parameter is set equal to the stimulation intensity and was drawn from a uniform distribution of 0–1 except when otherwise indicated. Simulated optogenetic stimulation was delivered to the network for 200 ms as was done in the macaque experiment. This duration was chosen in the macaque so that it matched visual stimulation duration for the macaque, for another purpose in that experiment. However, this aspect of the experiment—visual stimulation—was not incorporated into this model, whose goal was to gain insight into the dynamics of optogenetic stimulation.

## References

[1] Jazayeri M, Lindbloom-Brown Z, Horwitz GD. Saccadic eye movements evoked by optogenetic activation of primate V1. Nature neuroscience. 2012;15(10):1368–70. 10.1038/nn.3210 ; PubMed Central PMCID: PMCPMC3458167. 22941109 PMC3458167

[2] Jazayeri M, Remington E. Optogenetics Advances in Primate Visual Pathway. Neuron. 2016;90(1):8–10. 10.1016/j.neuron.2016.03.024 . 27054614

[3] Liu X, Ramirez S, Pang PT, Puryear CB, Govindarajan A, Deisseroth K, et al Optogenetic stimulation of a hippocampal engram activates fear memory recall. Nature. 2012;484(7394):381–5. 10.1038/nature11028 ; PubMed Central PMCID: PMCPMC3331914. 22441246 PMC3331914

[4] Pinto L, Goard MJ, Estandian D, Xu M, Kwan AC, Lee SH, et al Fast modulation of visual perception by basal forebrain cholinergic neurons. Nature neuroscience. 2013;16(12):1857–63. 10.1038/nn.3552 ; PubMed Central PMCID: PMCPMC4201942. 24162654 PMC4201942

[5] Sohal VS, Zhang F, Yizhar O, Deisseroth K. Parvalbumin neurons and gamma rhythms enhance cortical circuit performance. Nature. 2009;459(7247):698–702. 10.1038/nature07991 ; PubMed Central PMCID: PMCPMC3969859. 19396159 PMC3969859

[6] Tye KM, Deisseroth K. Optogenetic investigation of neural circuits underlying brain disease in animal models. Nature reviews Neuroscience. 2012;13(4):251–66. 10.1038/nrn3171 . 22430017 PMC6682316

[7] Nassi JJ, Avery MC, Cetin AH, Roe AW, Reynolds JH. Optogenetic Activation of Normalization in Alert Macaque Visual Cortex. Neuron. 2015;86(6):1504–17. 10.1016/j.neuron.2015.05.040 ; PubMed Central PMCID: PMCPMC4534089. 26087167 PMC4534089

[8] Han X, Qian X, Bernstein JG, Zhou HH, Franzesi GT, Stern P, et al Millisecond-timescale optical control of neural dynamics in the nonhuman primate brain. Neuron. 2009;62(2):191–8. 10.1016/j.neuron.2009.03.011 ; PubMed Central PMCID: PMCPMC2830644. 19409264 PMC2830644

[9] Sato TK, Hausser M, Carandini M. Distal connectivity causes summation and division across mouse visual cortex. Nature neuroscience. 2014;17(1):30–2. 10.1038/nn.3585 . 24241394 PMC5953407

[10] Carandini M, Heeger DJ. Normalization as a canonical neural computation. Nature reviews Neuroscience. 2012;13(1):51–62. 10.1038/nrn3136 ; PubMed Central PMCID: PMC3273486. 22108672 PMC3273486

[11] Reynolds JH, Heeger DJ. The normalization model of attention. Neuron. 2009;61(2):168–85. 10.1016/j.neuron.2009.01.002 ; PubMed Central PMCID: PMC2752446. 19186161 PMC2752446

[12] Carandini M, Heeger DJ, Movshon JA. Linearity and normalization in simple cells of the macaque primary visual cortex. The Journal of neuroscience: the official journal of the Society for Neuroscience. 1997;17(21):8621–44. . 9334433 10.1523/JNEUROSCI.17-21-08621.1997 PMC6573724

[13] Heeger DJ. Normalization of cell responses in cat striate cortex. Vis Neurosci. 1992;9(2):181–97. . 1504027 10.1017/s0952523800009640

[14] Mattis J, Tye KM, Ferenczi EA, Ramakrishnan C, O'Shea DJ, Prakash R, et al Principles for applying optogenetic tools derived from direct comparative analysis of microbial opsins. Nat Methods. 2011;9(2):159–72. 10.1038/nmeth.1808 ; PubMed Central PMCID: PMCPMC4165888. 22179551 PMC4165888

[15] Mateo C, Avermann M, Gentet LJ, Zhang F, Deisseroth K, Petersen CC. In vivo optogenetic stimulation of neocortical excitatory neurons drives brain-state-dependent inhibition. Curr Biol. 2011;21(19):1593–602. 10.1016/j.cub.2011.08.028 . 21945274

[16] Palmer LM, Schulz JM, Murphy SC, Ledergerber D, Murayama M, Larkum ME. The cellular basis of GABA(B)-mediated interhemispheric inhibition. Science. 2012;335(6071):989–93. 10.1126/science.1217276 . 22363012

[17] Urban-Ciecko J, Barth AL. Somatostatin-expressing neurons in cortical networks. Nature reviews Neuroscience. 2016;17(7):401–9. 10.1038/nrn.2016.53 . 27225074 PMC5635659

[18] Urban-Ciecko J, Fanselow EE, Barth AL. Neocortical somatostatin neurons reversibly silence excitatory transmission via GABAb receptors. Curr Biol. 2015;25(6):722–31. 10.1016/j.cub.2015.01.035 ; PubMed Central PMCID: PMCPMC4393017. 25728691 PMC4393017

[19] Stange A, Myoga MH, Lingner A, Ford MC, Alexandrova O, Felmy F, et al Adaptation in sound localization: from GABA(B) receptor-mediated synaptic modulation to perception. Nature neuroscience. 2013;16(12):1840–7. 10.1038/nn.3548 . 24141311

[20] Mann EO, Kohl MM, Paulsen O. Distinct roles of GABA(A) and GABA(B) receptors in balancing and terminating persistent cortical activity. The Journal of neuroscience: the official journal of the Society for Neuroscience. 2009;29(23):7513–8. 10.1523/JNEUROSCI.6162-08.2009 ; PubMed Central PMCID: PMCPMC4326656. 19515919 PMC4326656

[21] Self MW, Kooijmans RN, Super H, Lamme VA, Roelfsema PR. Different glutamate receptors convey feedforward and recurrent processing in macaque V1. Proceedings of the National Academy of Sciences of the United States of America. 2012;109(27):11031–6. 10.1073/pnas.1119527109 ; PubMed Central PMCID: PMCPMC3390882. 22615394 PMC3390882

[22] Herrero JL, Gieselmann MA, Sanayei M, Thiele A. Attention-induced variance and noise correlation reduction in macaque V1 is mediated by NMDA receptors. Neuron. 2013;78(4):729–39. 10.1016/j.neuron.2013.03.029 ; PubMed Central PMCID: PMC3748348. 23719166 PMC3748348

[23] Carlen M, Meletis K, Siegle JH, Cardin JA, Futai K, Vierling-Claassen D, et al A critical role for NMDA receptors in parvalbumin interneurons for gamma rhythm induction and behavior. Molecular psychiatry. 2012;17(5):537–48. 10.1038/mp.2011.31 ; PubMed Central PMCID: PMCPMC3335079. 21468034 PMC3335079

[24] Buchanan KA, Blackman AV, Moreau AW, Elgar D, Costa RP, Lalanne T, et al Target-specific expression of presynaptic NMDA receptors in neocortical microcircuits. Neuron. 2012;75(3):451–66. 10.1016/j.neuron.2012.06.017 ; PubMed Central PMCID: PMCPMC3657167. 22884329 PMC3657167

[25] Zhu Y, Qiao W, Liu K, Zhong H, Yao H. Control of response reliability by parvalbumin-expressing interneurons in visual cortex. Nature communications. 2015;6:6802 10.1038/ncomms7802 . 25869033

[26] Sundberg KA, Mitchell JF, Gawne TJ, Reynolds JH. Attention influences single unit and local field potential response latencies in visual cortical area V4. The Journal of neuroscience: the official journal of the Society for Neuroscience. 2012;32(45):16040–50. 10.1523/JNEUROSCI.0489-12.2012 ; PubMed Central PMCID: PMCPMC3711872. 23136440 PMC3711872

[27] Cohen MR, Maunsell JH. Attention improves performance primarily by reducing interneuronal correlations. Nature neuroscience. 2009;12(12):1594–600. Epub 2009/11/17. 10.1038/nn.2439 ; PubMed Central PMCID: PMC2820564. 19915566 PMC2820564

[28] Mitchell JF, Sundberg KA, Reynolds JH. Spatial attention decorrelates intrinsic activity fluctuations in macaque area V4. Neuron. 2009;63(6):879–88. Epub 2009/09/26. 10.1016/j.neuron.2009.09.013 ; PubMed Central PMCID: PMC2765230. 19778515 PMC2765230

[29] Abbott LF, Varela JA, Sen K, Nelson SB. Synaptic depression and cortical gain control. Science. 1997;275(5297):220–4. . 8985017 10.1126/science.275.5297.221

[30] Chance FS, Abbott LF, Reyes AD. Gain modulation from background synaptic input. Neuron. 2002;35(4):773–82. . 12194875 10.1016/s0896-6273(02)00820-6

[31] Olsen SR, Wilson RI. Lateral presynaptic inhibition mediates gain control in an olfactory circuit. Nature. 2008;452(7190):956–60. 10.1038/nature06864 ; PubMed Central PMCID: PMCPMC2824883. 18344978 PMC2824883

[32] Adesnik H, Bruns W, Taniguchi H, Huang ZJ, Scanziani M. A neural circuit for spatial summation in visual cortex. Nature. 2012;490(7419):226–31. 10.1038/nature11526 ; PubMed Central PMCID: PMC3621107. 23060193 PMC3621107

[33] Nassi JJ, Gomez-Laberge C, Kreiman G, Born RT. Corticocortical feedback increases the spatial extent of normalization. Frontiers in systems neuroscience. 2014;8:105 10.3389/fnsys.2014.00105 ; PubMed Central PMCID: PMCPMC4039070. 24910596 PMC4039070

[34] Abilez OJ, Wong J, Prakash R, Deisseroth K, Zarins CK, Kuhl E. Multiscale computational models for optogenetic control of cardiac function. Biophys J. 2011;101(6):1326–34. 10.1016/j.bpj.2011.08.004 ; PubMed Central PMCID: PMCPMC3177076. 21943413 PMC3177076

